# Selective sweep sites and SNP dense regions differentiate *Mycobacterium bovis* isolates across scales

**DOI:** 10.3389/fmicb.2022.787856

**Published:** 2022-09-07

**Authors:** Noah Legall, Liliana C. M. Salvador

**Affiliations:** ^1^Interdisciplinary Disease Ecology Across Scales Research Traineeship Program, University of Georgia, Athens, GA, United States; ^2^Institute of Bioinformatics, University of Georgia, Athens, GA, United States; ^3^Center for the Ecology of Infectious Diseases, University of Georgia, Athens, GA, United States; ^4^Department of Infectious Diseases, College of Veterinary Medicine, University of Georgia, Athens, GA, United States

**Keywords:** comparative genomics, *Mycobacterium bovis*, host range, geographic location, population clusters, selective sweeps, SNP dense regions, ecological scales

## Abstract

*Mycobacterium bovis*, a bacterial zoonotic pathogen responsible for the economically and agriculturally important livestock disease bovine tuberculosis (bTB), infects a broad mammalian host range worldwide. This characteristic has led to bidirectional transmission events between livestock and wildlife species as well as the formation of wildlife reservoirs, impacting the success of bTB control measures. Next Generation Sequencing (NGS) has transformed our ability to understand disease transmission events by tracking variant sites, however the genomic signatures related to host adaptation following spillover, alongside the role of other genomic factors in the *M. bovis* transmission process are understudied problems. We analyzed publicly available *M. bovis* datasets collected from 700 hosts across three countries with bTB endemic regions (United Kingdom, United States, and New Zealand) to investigate if genomic regions with high SNP density and/or selective sweep sites play a role in *Mycobacterium bovis* adaptation to new environments (e.g., at the host-species, geographical, and/or sub-population levels). A simulated *M. bovis* alignment was created to generate null distributions for defining genomic regions with high SNP counts and regions with selective sweeps evidence. Random Forest (RF) models were used to investigate evolutionary metrics within the genomic regions of interest to determine which genomic processes were the best for classifying *M. bovis* across ecological scales. We identified in the *M.* bovis genomes 14 and 132 high SNP density and selective sweep regions, respectively. Selective sweep regions were ranked as the most important in classifying *M. bovis* across the different scales in all RF models. SNP dense regions were found to have high importance in the badger and cattle specific RF models in classifying badger derived isolates from livestock derived ones. Additionally, the genes detected within these genomic regions harbor various pathogenic functions such as virulence and immunogenicity, membrane structure, host survival, and mycobactin production. The results of this study demonstrate how comparative genomics alongside machine learning approaches are useful to investigate further the nature of *M. bovis* host-pathogen interactions.

## Introduction

Bovine tuberculosis (bTB) is a livestock disease caused by the transmission of the bacterial pathogen *Mycobacterium bovis* (*M. bovis*), and has wide ranging impacts on agriculture, economics, and human health (Palmer et al., 2012; Palmer, 2013). *M. bovis* is a member of the *Mycobacterium tuberculosis* complex (MTBC), which is a genetically related group (99% similarity) of Mycobacterium species that can cause tuberculosis within vertebrate host-species, including humans (Brosch et al., 2002). *Mycobacterium tuberculosis* is the main causative agent of human associated tuberculosis, while other lineages are defined in vertebrate hosts such as *M. africanum*, *M. canettii*, *M. microti*, *M. bovis*, *M. caprae*, *M. pinnipedii*, *M. mungi*, *M. orygis*, and *M. suricattae* (Patané et al., 2017; Lekko et al., 2020). The wide range of MTBC members is hypothesized to be mainly due to the divergence of certain lineages from an ancestral species *Mycobacterium canettii* through multiple insertion or deletion mutations (Gutierrez et al., 2005; Patané et al., 2017).

*Mycobacterium bovis* has been identified as having the widest mammalian host range to date compared to other members of the MTBC (Palmer, 2013). This allows *M. bovis* to transmit between species much more easily, such that if close spatial proximity between wild and domestic animals can occur through direct contact (infected individuals and/or carcasses) or indirect contact (contaminated soil or water resources), frequent spillover events to and/or from wildlife can occur (Palmer, 2013; Gormley and Corner, 2018). Certain regions in the United States of America (USA), New Zealand (NZ), and the United Kingdom (UK) are endemic for bTB, in which certain wildlife species have transitioned from novel spillover hosts to reservoirs of infection, defined as populations that can maintain a pathogen and transmit it to a target population (Haydon et al., 2002; Viana et al., 2014; Hallmaier-Wacker et al., 2017). The consistent contact between a wildlife reservoir and livestock can lead to frequent spillback of *M. bovis* into livestock populations, jeopardizing existing control measures to mitigate the disease. These dynamics exacerbate the agricultural and economic impacts bTB has on society, with contemporary estimates of 50 million cattle worldwide infected and costing farmers $3 billion annually (Palmer, 2013). However, only a few species have successfully transitioned from novel spillover host to reservoir of infection, suggesting that there exist certain factors that limit the ability of *M. bovis* to adapt to specific hosts. Understanding how these fundamental processes occur in order for hosts to transition from spillover to adapted host populations would contribute to our understanding of pathogen-host interactions and inform disease control measures in order to mitigate the impacts of *M. bovis* transmission.

The use of next-generation sequencing (NGS) to study pathogen genomics has revolutionized the field of *M. bovis* molecular epidemiology. Detection of genomic variations among multiple samples have allowed researchers to characterize *M. bovis* population structure (Müller et al., 2009; Berg et al., 2011; Smith et al., 2011; Rodriguez-Campos et al., 2012; Reis and Cunha, 2021; Rodrigues et al., 2021), sources and chains of transmission (McCluskey et al., 2014; Milian-Suazo et al., 2016; Orloski et al., 2018; Loiseau et al., 2020; Zimpel et al., 2020; Tonder et al., 2021; Rossi et al., 2022), and the role of host-species in the transmission process (Biek et al., 2012; Crispell et al., 2017, 2019, 2020; Salvador et al., 2019; Akhmetova et al., 2021). Authors from Fuente et al. (2015) studied how single nucleotide polymorphisms (SNPs) and gene presence/absence contributed to lesion scores on three *M. bovis* isolates in an attempt to associate genomic changes with phenotype differences in *M. bovis*. The results highlighted that there were key differences between the presence and absence of genes that were associated with host–pathogen interactions and resulting virulence among hosts. However, it is still unclear if there are *M. bovis* genomic variations across different environments (e.g., host-associated populations of *M. bovis* or geographical locations) and if they play a specific role in the adaptation process to those environments. Genomic analyses like these would unravel specific evolutionary forces responsible for *M. bovis* adaptation to particular environments (Allen, 2017; Patané et al., 2017; Sheppard et al., 2018) and improve our understanding of *M. bovis* evolution across different ecological scales.

One avenue to explore the genomic factors that impact *M. bovis* adaptation is to investigate genomic regions of interest that potentially harbor signatures of *M. bovis* evolution. For instance, within different biological populations of organisms, a highly beneficial mutation that is introduced into the population can quickly become the most common allele, leading to a lack of variation at that location (Stephan, 2019). The site of this beneficial mutation can be in linkage disequilibrium with neutral flanking SNPs, meaning that the variation at these neighboring SNP sites would be non-random and correlated to the variation up and downstream of a beneficial site (Alachiotis et al., 2012). This signature, defined as a ‘selective sweep’ can highlight important genes linked with *M. bovis* adaptation to new environments. Additionally, bacterial genomes can possess regions with a higher number of mutations than expected if analyzed genome wide. Regions of high SNP density might indicate sites of elevated mutation rates that provide short term fitness advantages when adapting to a new ecological niche. This phenomena, often defined as hypermutation, is common in pathogenic bacteria that must adapt to newly encountered stressors such as antibiotic resistance and novel hosts (Swings et al., 2017; Mehta et al., 2019). A deeper investigation into *M. bovis* genomic regions from isolates extracted from multiple hosts and geographic locations can provide an opportunity to determine *M. bovis* evolutionary processes that might confer adaptive advantages. In this study, we conduct a comparative genomic study of *M. bovis* isolates collected from livestock and wildlife host-species from three bTB endemic regions around the world. We use WGS data to create a high-resolution genomic dataset with spatial and host phenotypic information. Our objectives for this study are to: (a) identify *M. bovis* genomic signatures; (b) investigate the relationship of these genomic signatures across different scales (geographical, host-species and sub-populations scales); and (c) determine sets of genes associated with the *M. bovis* genomic regions of interest and/or the different scales.

## Materials and methods

### Data description

In this study, we downloaded a total of 700 *Mycobacterium bovis* whole-genomes from the National Center for Biotechnology Information (NCBI) Sequence Read Archive (SRA). These isolates originated from three previous studies focusing on *M. bovis* evolutionary dynamics and cross-species transmission in NZ (PRJNA363037; Crispell et al., 2017), UK—Woodchester Park (PRJNA523164; Crispell et al., 2019), and in the USA—Michigan (PRJNA251692; Salvador et al., 2019), respectively. Metadata associated with these isolates included geographic location and host species from which *M. bovis* samples were extracted (11 in total): six in NZ [stoat, *Mustela erminea*; porcine, *Sus scrofa*; cervine (various NZ cervine species unknown); cattle, *Bos taurus*; brushtail possum, *Trichosurus vulpecula*; and ferret, *Mustela furo*], two in the UK (cattle, *Bos taurus* and the Eurasian badger, *Meles meles*), and three in the USA (white-tailed deer, *Odocoileus virginianus*; elk, *Cervus canadensis*; and cattle, *Bos taurus*). Although there was only one *M. bovis* isolate from a stoat host, it was kept in the dataset to assist in further analyses of geographic and subpopulation structure. Bioinformatic statistics associated with the isolates are shown in Supplementary Table 1.

### Data processing

To improve the quality from the paired-end sequences, we used fastp v0.22 (Chen et al., 2018) to remove adapter sequences and low-quality ends. We used a sliding window approach (size 4 bp) to trim reads with window average quality below 30. Additionally, we filtered out all reads less than 15 bp. Once all the reads were processed, we mapped them against the *M. bovis* reference genome AF2122/97 (NC_002945.4, Genbank accession code PRJNA89) using the Burrows-Wheeler Aligner (BWA) software (Li and Durbin, 2009). We removed duplicated reads using Picard v2.0.1 (Broadinstitute/Picard, 2021/2014) to limit the impact of non-unique or erroneous reads on the SNP calling process. We performed Variant calling using Freebayes v1.3.5 (Garrison and Marth, 2012), and we kept detected SNPs for the downstream analysis if they possessed (i) a phred-quality score (QUAL) above 20, (ii) a mapping quality (MQ) above 59, (iii) did not fall within gene regions that coded for *pe, pe/PGRS and ppe* genes (a family of redundant sequence genes that are difficult to work with *in-silico*; Sampson, 2011; Delogu et al., 2017), and (iv) were more than 500 bp from insertion or deletion mutation (INDEL) regions (this is an extra measure to reduce impacts in the alignment that can lead to false positive detections). Furthermore, the isolates used in this study were sequenced either on a HiSeq or MiseqIllumina platform, and we compared specific bioinformatic metrics (read depth, number of SNPs, SNP depth, and bp depth) between isolates to check if the choice of sequencing platform played a role in SNP detection and quality. A SNP alignment was created using BCFtools v1.13 (Li, 2011) ‘consensus’ command and snp-sites v2.5.1 (Page et al., 2016) to integrate the detected SNPs into a copy of the *M. bovis* reference genome.

### Phylogenetic inference

Evolutionary relationships among *M. bovis* isolates were investigated using the Maximum Likelihood software IQtree v2.1.4 (Minh et al., 2020) with the *M. bovis* SNP alignment used as input. IQTree uses the ModelFinder approach to determine the nucleotide substitution model that best fits the data based on the Bayesian Information Criteria (BIC) comparison between substitution models (Kalyaanamoorthy et al., 2017). After inferring the substitution model, IQtree ran 1,000 bootstrap iterations in order to provide a measure of internal node support ranging from 0 to 100 (Hoang et al., 2018).

To determine if the existence of potential regions with elevated densities of base substitutions in the *M. bovis* genomes has any effect on the resolution of their phylogeny and nodal support, we compared a phylogeny produced with the original SNP alignment data with the one generated by the Gubbins software (Croucher et al., 2015). Each phylogeny was assessed based on the proportion of highly supported internal nodes (nodal support). After choosing the phylogeny with the best nodal support, additional comparisons between phylogenies were made based on different rooting strategies such as using the *M. bovis* reference (AF2122/97, NC_002945.4, Genbank accession code PRJNA89) as an outgroup, Minimal Ancestor Deviation (Tria et al., 2017), and Midpoint rooting (Kinene et al., 2016). The best rooting strategy was based on comparisons of Robinson Foulds distance between the different rooted phylogenies, which is a popular metric to ascertain the number of operations required to convert the topology of one tree to another (Pattengale et al., 2007). If this value was large, then the two trees being compared are highly dissimilar. After assessing which phylogeny was more representative of the evolutionary history between isolates, that phylogeny was used to simulate an *M. bovis* alignment that will be used to generate null distributions of SNP density and selective sweep regions (sections below).

### Population structure

In order to determine *M. bovis* population structure, we used fastBAPS v1.0.4 (Tonkin-Hill et al., 2019), a model-based clustering approach, to determine clusters of related sequences present in the genetic alignment. This software utilizes a Bayesian hierarchical clustering approach that handles large alignments and quickly discerns sub-populations. As input, fastBAPS received the SNP only alignment and utilized the optimized symmetric prior with the assumption that all allele frequencies at a SNP site are equivalent (making it analogous to a uniform-like prior). The sub-population clusters were extracted directly from the output of fastBAPS.

### Simulated *Mycobacterium bovis* alignment

**Figure 1:**
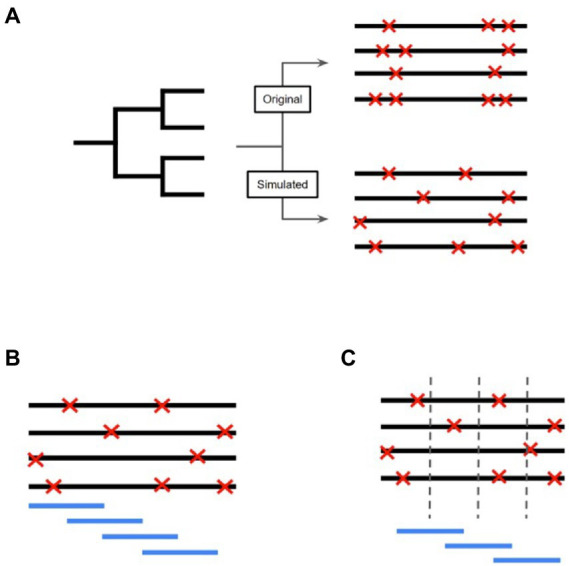
Workflow to detect SNP Dense Regions and Selective Sweep Regions in the *Mycobacterium bovis* genome. **(A)** The inferred phylogeny created using the original data is used as the input for Alisim to simulate an alignment based on the topology of the *M. bovis* maximum likelihood trees. This simulated alignment will differ from the original alignment since the SNP positions (symbolized by red ‘X’s) will be included randomly and independently. **(B)** After obtaining the simulated alignment, a sliding window approach is used to identify SNPs present in each window. The window starts at the beginning of the alignment, and moves across the entire genome by a defined step size. **(C)** A window approach was also used to investigate regions of selective sweeps. The window was centered on a predetermined nucleotide coordinate, and the amount of linkage disequilibrium was compared upstream and downstream of the coordinate.

To investigate if certain regions in the *M. bovis* genome have significantly higher values of either SNP density or selective sweep sites, a probabilistic hypothesis test was used to find highly significant genomic regions. For that, we produced a simulated alignment from the original alignment with the tool Alisim using the same alignment length, nucleotide substitution model, and phylogenetic tree topology (created from the previously computed Maximum Likelihood tree; Ly-Trong et al., 2022). Alisim next created a random sequence based on the previous specifications and then simulated nucleotide substitutions that were independently added while also conforming to the phylogenetic topology, resulting in a simulated alignment output. From the simulated alignment, we performed two separate sliding window analyses for calculating SNP dense regions and Selective Sweep regions, respectively. We captured the number of SNPs for the SNP density analysis and the support for selective sweep events in the selective sweep analysis. The data generated from the two separate sliding window analyses were then used to create two null distributions that were used to perform hypothesis testing for the SNP dense and selective sweep regions in the original data. Significance was determined by performing a hypothesis test on each window, where the probability of calculating a certain metric in the sliding window from the original data was compared to the probability of seeing the same metric in the null distribution (Figure 1A).

### SNP dense regions

To find SNP dense regions in the genome alignment, we implemented a SNP counting approach that used a sliding window to find regions with significantly higher SNP counts than what would be expected by chance (Figure 1B). A combination of sliding window size (w) and step size (s) values, where *w* = {100, 500, 1,000, 2,500, 5,000}, *s* = {50, 100, 300, 500, 1,000}, and *w* > *s*, was used to determine the best combination of parameters to the highest number of SNPs in windows that were found to be significant (Tajima, 1991). Once the optimal window and step lengths were inferred, we used those lengths for each region in the genome to identify high SNP counts. Significance was determined by performing a hypothesis test on each window, where the probability of finding a certain number of SNPs in a window was based on the simulated alignment null distribution. The higher the number of SNPs present in a window, the lower the probability that this window evolved due to conventional means. Our implemented approach was simpler than other tools that are meant to find regions with high SNP density. For example, pre-existing software tools, such as Gubbins, detect regions with high SNP counts by first allowing a sliding window to take lengths between 0.1 to 10 kb to identify regions with at least 10 SNPs. Gubbins methodology also incorporates additional hypothesis tests to find both regions with high SNP counts and putative regions of homologous recombination. Our implementation of high SNP regions ensured that we were extracting all of the genomic regions with high SNP counts and not only regions that had high SNP counts and putative support for homologous recombination (which would make us miss some of the high SNP counts regions).

The null distribution should have had characteristics of a Poisson distribution since we were counting the number of SNPs in a certain window size. Therefore, Poisson distribution parameters for this null distribution were estimated using the R package *fitdistrplus* based on a maximum likelihood method (R Core Team, 2022; Delignette-Muller and Dutang, 2015). For each window that was analyzed, we performed a statistical hypothesis test to determine the probability that the number of SNPs in a window came from the null distribution. Since multiple tests were required for the alignment, to reduce the number of false-positive regions, we used a Bonferroni corrected *p*-value (Dunn, 1961). If the probability of the number of SNPs based on the null distribution was lower than the Bonferroni corrected *p*-value {*p*-value/(the number of tests)}, then that window was recorded as having a significantly higher number of SNPs than expected by chance. The combination of *w* and *s* that produced the highest number of significant results was used for subsequent analyses. We merged the overlapping windows to highlight regions that had high numbers of SNPs throughout using BEDtools (Quinlan and Hall, 2010). These discrete regions were then labelled as SNP dense regions (SDRs).

### Selective sweep regions

To identify the sites that were the center of the selective sweep regions, we used the tool OmegaPlus v3.03 (Alachiotis et al., 2012) to determine the value omega, which summarizes the extent of linkage disequilibrium (LD) upstream and downstream of a pre-determined genomic position in the *M. bovis* genome alignment (Figure 1C). The higher the omega value, the higher the possibility that the genomic position is within the proximity of a selective sweep. We computed the omega values for 5,000 equidistant positions within the simulated alignment. Similar to the protocol employed for detecting SNP dense regions, we first characterized the null distribution of omega values as a gamma distribution since the values started at zero, were continuous, and theoretically could have been unbounded. After filtering out the positions that had the value of zero, we again used the *fitdistrplus* package to estimate the gamma distribution parameters based on the maximum likelihood method. For each window that was analyzed, we performed a statistical hypothesis test with Bonferroni corrected *p*-values to determine the probability that the evidence for a selective sweep coming from that region was from the null distribution. To determine the appropriate maximum window size, we computed the number of significant results (*n*) using multiple window sizes *w* = {150, 300, 500, 750, 1,000, 1,500, 3,000, 5,000, and 10,000}, and divided the number of significant results by *w*. Since the calculation of the omega statistic consistently increases as *w* increases, dividing the number of successful regions by *w* provided a way to find window sizes that did not add extra significant results to the analysis. The maximum value of *w* was determined by finding the window size that maximized the calculation (number of significant windows)/(window size length). We merged the overlapping windows to highlight genomic regions that possessed high evidence for selective sweep events using BEDtools. These discrete regions were then labelled as selective sweep regions (SSRs).

### Random forest modeling

**Figure 2:**
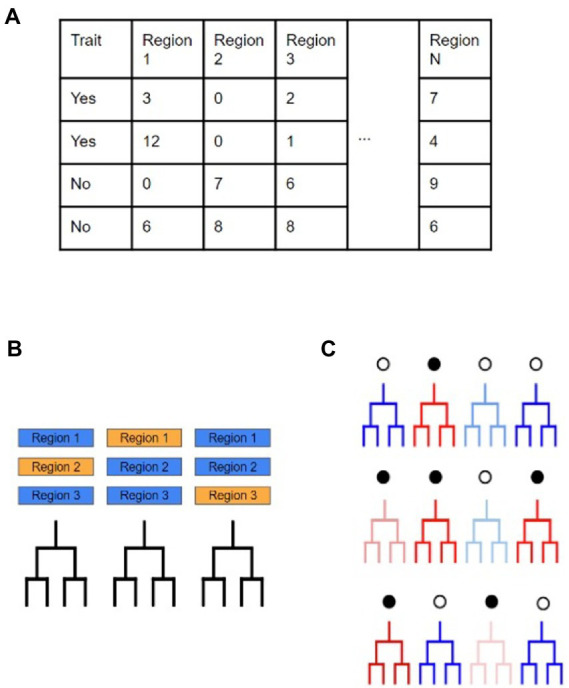
Workflow to determine evolutionary predictors for *M. bovis* across scales. **(A)** Here the dataset is divided based on a binary trait of ‘yes’ or ‘no’ with accompanying predictor variables that may provide support in making classifications on the trait. In this study, these predictors are as follows: the number of SNPs in a SNP dense region, the number of INDELs in a SNP dense region, the number of SNPs in a selective sweep region, the number of SNPs in coding regions, the number of SNPs in non-coding regions, the number of INDELs in coding regions, the number of INDELs in non-coding regions, and the genome wide GC%. **(B)** Random Forest models use multiple, uncorrelated decision trees created from randomly subset predictors of the original data. Due to the individual trees being unrelated to each other, their combined predictive ability leads to excellent classification ability. **(C)** Since each individual decision tree is created from random subsets of the predictors, the accuracy of individual trees will vary greatly (red and blue represent low and high accuracy, respectively). The amount of increase and/or decrease in model accuracy when a specific predictor is included is determined by tracking which predictors are included in the simple decision trees.

After finding the genomic regions with statistical support for increased SNP occurrence and selective sweeps, we measured the impact that evolution in these regions has on being able to differentiate isolates based on their ecological grouping. Random Forest models supported by the random Forest package, were used to perform classification of isolates based on evolutionary metrics to elucidate if SDRs and/or SSRs were important individual predictors in correctly classifying the *M. bovis* data (Figure 2; Breiman, 2001). Specifically, we used the following data as predictors in our models: (i) SDR number of SNPs, (ii) SDR number of INDELs, and (iii) SSR number of SNPs. To measure how the genomic regions we found compare to general characteristics of the *M. bovis* genomes, we also included other genomic metrics of the isolates in our analysis. GC content was calculated directly from the full *M. bovis* alignment, whereas number of non-coding SNPs, number of coding SNPs, number of non-coding INDELs, and number of coding INDELs were extracted directly from each isolate’s variant calling format (VCF) file. Prior to model fitting, predictors with a high amount of correlation with other predictors were removed in order to limit redundant data using the R *caret* package (see Supplementary File 3; Kuhn, 2008). Model performance was calculated based on the overall accuracy in predicting the correct *M. bovis* isolate membership for particular groupings. A predictor’s importance was measured through its mean decrease in accuracy (MDA), with higher values indicating that models which did not incorporate the predictor suffered by the indicated number of accuracy points. For each random forest analysis, we ordered predictors based on their MDA and recorded the top 20 most important ones. Certain analyses called for predictors to be compared across different models, such as comparing predictor importance across ecological scales, global versus local cattle hosts, and wildlife reservoir versus livestock hosts. For these analyses, Venn diagrams were constructed to observe which predictors were shared or unique amongst the models. Additionally, predictors that saw sharp increases or decreases in importance when compared across models were recorded.

### Gene functions associated with models

After key genomic regions were identified from each random forest model, we used BEDtools to identify sets of genes that were present within important SDRs and SSRs. In particular, we highlight genes that have been catalogued in previous research as playing a role in host pathogen interactions in mycobacterial pathogens.

## Results

### Phylogenetic reconstruction of *Mycobacterium bovis* isolates

Whole-genome sequencing of the 700 *M. bovis* isolates from studies implemented in NZ, UK, and the USA identified a total of 6,847 SNPs (Supplementary Table 1). Even though the genomes were sequenced on different platforms, in the 692 samples that were sequenced on Illumina HiSeq 2500 (197) and Illumina MiSeq (495), the number of SNPs, depth per base pair, and average depth per genome did not deviate much across platforms (Supplementary Figure 1). Additionally, the number of SNPs that were detected in coding versus non-coding regions of the genome shared similar distributions between all isolates sequenced on the differing platforms (Supplementary Figure 2), suggesting that the choice of sequencing technology did not influence the downstream results. Isolates sequenced on a NextSeq platform (8) were not included in the violin plots constructed for Supplementary Figures S1, S2 due to an insufficient number of isolates characterized with this platform.

**Figure 3:**
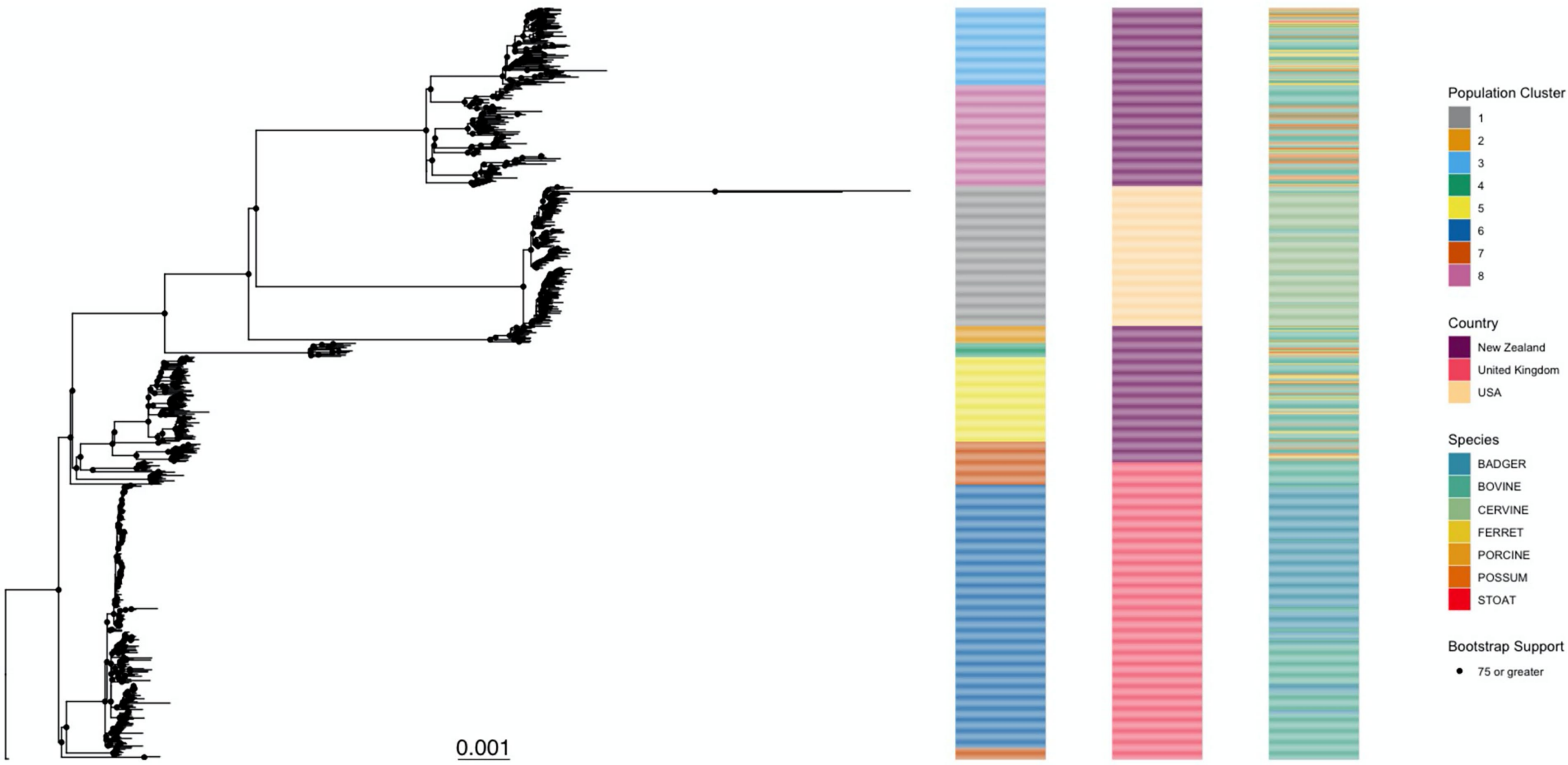
*Mycobacterium bovis* maximum likelihood phylogenetic tree. This tree was inferred from the 6,847 SNPs of 700 *Mycobacterium bovis* isolates extracted from three independent studies in endemic regions: New Zealand, United Kingdom, and the United States of America. The TPM2+F+R4 substitution model was the one with best statistical support (by Bayesian Information Criteria). Ultrafast bootstrap support for the internal nodes is indicated by a black circle, its presence indicating that the node has support of >75%. The phylogeny is further annotated by the taxa’s membership in a population cluster (left), country of origin (middle), and host species (right). The tree was rooted using the *M. bovis* reference genome AF2122/97 as an outgroup.

When comparing the phylogenetic trees created from the original alignment and the one outputted by Gubbins, we found that there was a Robinson Foulds distance of 592 between the two trees, meaning that one tree would need 592 changes to be converted to the other. The Gubbins phylogeny possessed better nodal support (75.7% of internal nodes with bootstrap value of 75 or above) than the phylogeny produced from the original alignment (70.1% of internal nodes with bootstrap value of 75 or above; Supplementary Table 2), which suggests that it provides a better approximation of the evolutionary history between the *M. bovis* isolates in this dataset, and therefore, was used both for the rooting method comparison and for simulating the *M. bovis* alignment. The best rooting strategy for the phylogeny was the Outgroup rooting method [using the reference AF2122/97 (NC002945.4)], since it led to fewer changes in the topology (12 and 14) when compared to the Midpoint and MAD rooting strategies (Supplementary Figure 3, Supplementary Table 3). The substitution model that best fit the data was the TPM2+F+R4. The *M. bovis* phylogeny produced clustering patterns that were distinguishable based on geographic region (Figure 3). The population clustering analysis identified eight distinct clusters that matched closely with geographical locations. For instance, Cluster 1 had the same isolates as the USA group. Clusters 6 and 7 were subpopulations of the UK group, and NZ isolates contained the remaining population clusters (Clusters 2, 3, 4, 5, and 8). The population clusters did not show a visible pattern based on host species, indicating that the estimated *M. bovis* population clusters are determined mostly by geographical location.

### SNP density and selective sweep region identification

**Figure 4:**
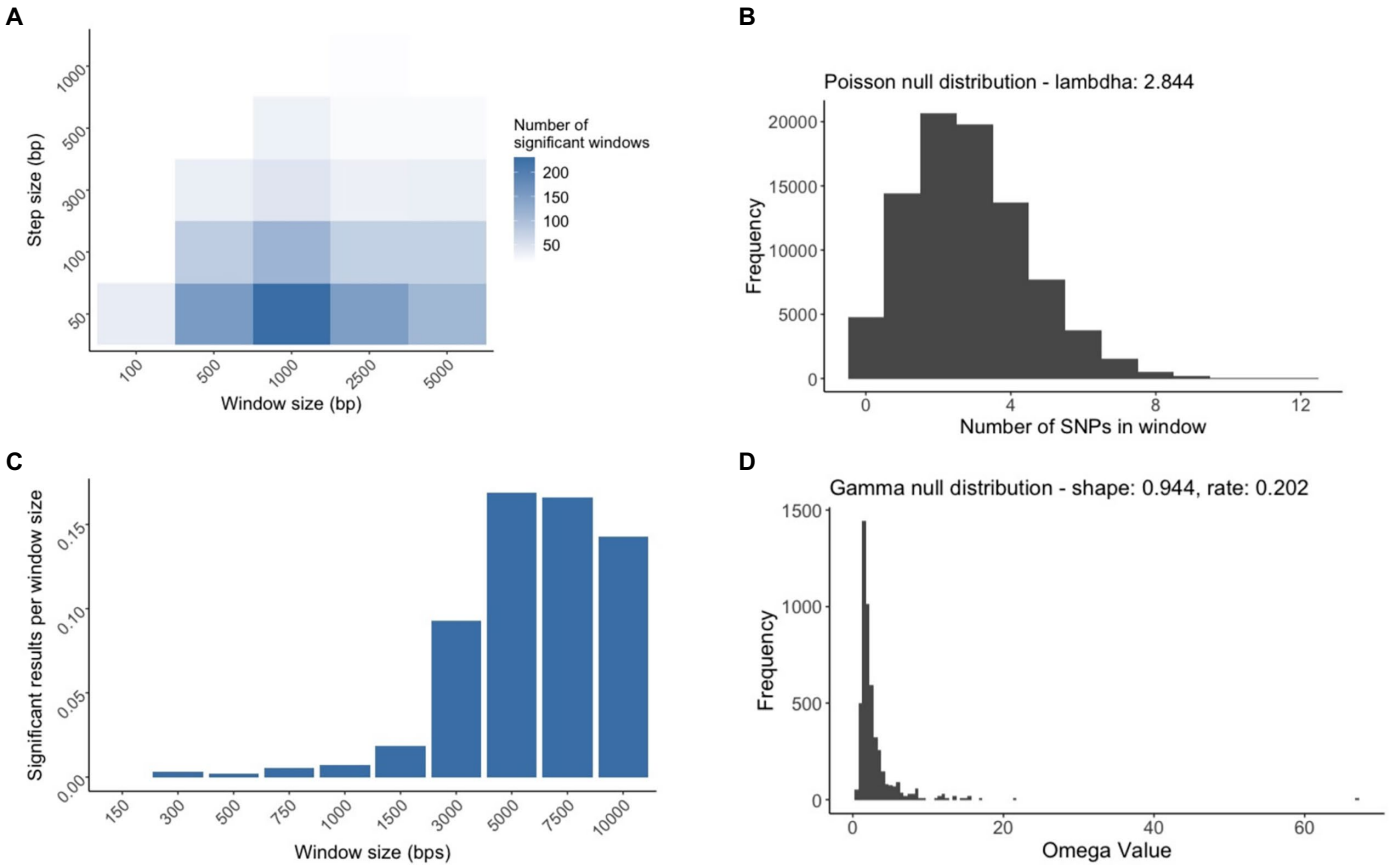
The process of creating the null distributions used for SNP Dense Regions (SDRs) and Selective Sweep Regions identification (SSRs). **(A)** Multiple sliding window analyses were conducted to find the window size and step size whose combination maximized the number of significant windows. The white to blue gradient represent the indicated increased number of significant results. **(B)** The genome wide hypothesis test for SDRs relied on a window size of 1,000 bp and a step size of 50 bp to generate a Poisson null distribution for subsequent tests. **(C)** Multiple selective sweep analyses were conducted by varying the window size to find the size that maximized the number of significant results normalized by the window length. **(D)** The genome wide hypothesis test for SSRs relied on the window size of 5,000 bp to generate a Gamma null distribution for subsequent tests.

The simulated *M. bovis* alignment was produced using the TPM2+F+R4 model, which was also the best supported model. For the SNP Density analysis, we determined that the combination of a window size of 1,000 bp and a step size of 50 bp were the parameters that identified the most significant windows (Figure 4A). In each window, we counted the number of SNPs that were within that window and represented that data as a Poisson distribution. We estimated the Poisson distribution parameter lambda to be 2.84, which suggests that in our simulated alignment, we can expect to have 2.84 SNPs in a window on average with a variance of 2.84 (Figure 4B). For the selective sweep analysis, we identified that a window size of 5,000 bp was needed to maximize the number of significant results (Figure 4C), and similarly we estimated the parameters of the Gamma null distribution to be 0.944 (the shape parameter estimates the typical omega value in a window), and 0.202 (the scale parameter estimate that variance of the omega value), respectively. Based on the inferred parameter values for both null distributions, the genome-wide hypothesis tests computed the probability of finding the amount of SNPs/evidence of a selective sweep within the tested window. After we merged the overlapping significant windows, we determined 14 unique SDRs and 132 unique SSRs (Supplementary Tables 4, 5). A few SDRs and SSRs overlapped regions with regions containging *pe* or *ppe* genes, but since the SNPs falling within these genes were removed from the analysis, these genes did not contribute to the identification of SDRs or SSRs.

### Random forest analysis

#### Shared and unique regions across scales

Three random forest models were computed for each individual scale present in our data: country of origin, sub-population, and host-species. Models presented different accuracy depending on the scale in focus. For the host species model, the model accuracy to classify *M. bovis* hosts was low for the majority of the host species represented in the data, especially within phylogenetic clades where multiple host species were present (low host-species structure). However, the model achieved high accuracy in classifying isolates from the UK as being either from cattle or badger hosts, with accuracies of 94.5 and 94.6%, respectively.

**Figure 5:**
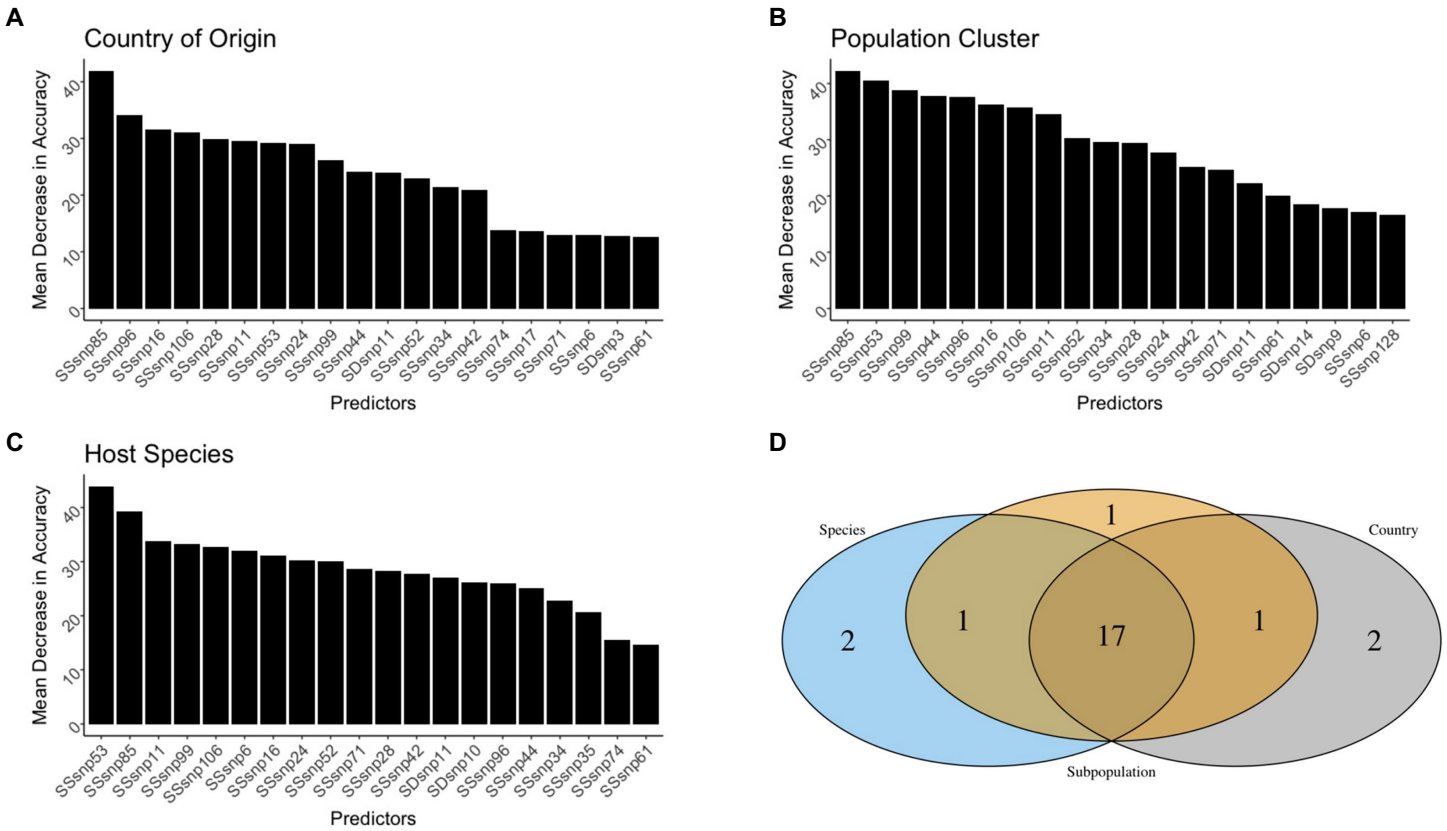
The top 20 predictors across the different scales. The predictors were presented for the Country of origin **(A)**, Population cluster **(B)**, and Host species **(C)** random forest models. A Venn diagram was used to identify predictive genomic regions that were shared and unique across models **(D)**.

For each model, SSRs achieved the highest importance rankings for model classification, with a few SDRs ranking as some of the top 20 predictors for each model. As for the other genomic evolutionary metrics (such as INDELS and GC content), which were included in the model to determine how important SSRs or SDRs were, they were not included amongst the top 20 predictors (Figures 5A–C).

Out of all the regions used in the random forest analysis, only 16 SSRs and one SDR were shared as the top 20 most important variables across all the models (Figure 5D). It was rare for the top SSRs and SDRs of the three random forest models to be shared by two models only. For instance, country-of-origin and population cluster, as well as population cluster and host species, shared one region amongst their top 20 predictors, but zero regions were shared between the country of origin and species models. Each individual model also had at least two SSRs/SDRs that were uniquely important for correct classification (Figure 5).

**Figure 6:**
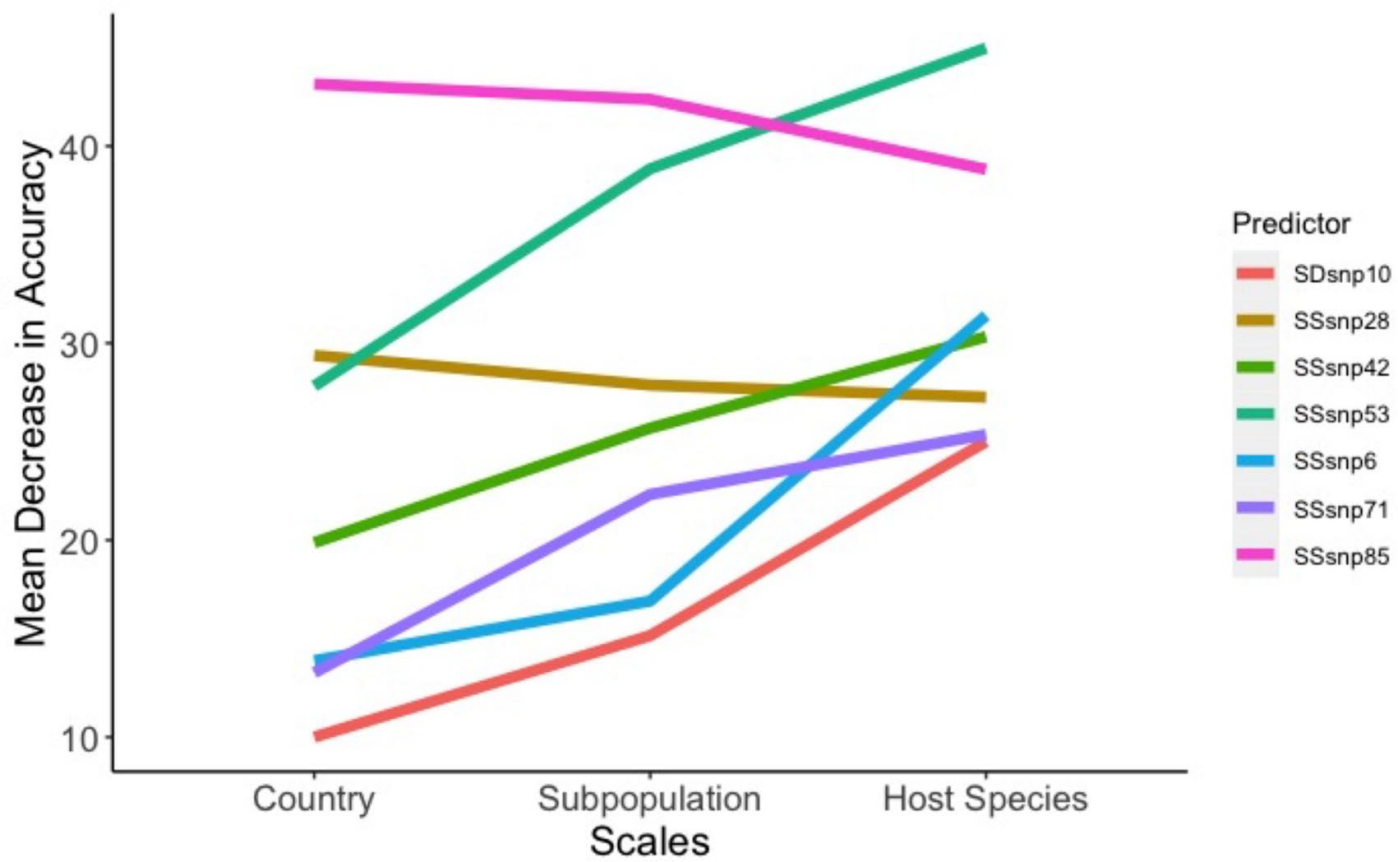
Factors that affect model accuracy across scales. The seven regions between the ‘across-scales model’ that increase or decrease the model accuracy as the scales move from general (Country of origin) to less specific (Host species).

Amongst all predictors in our models, only four SSRs and one SDR helped improve the model accuracy as the scales went from being general (country-of-origin) to more specific (host-species). Conversely, only two SSRs were found to be of decreased importance (Figure 6). SSR53 was an important region regardless of the model, with MDAs for country of origin, population clustering, and species being 27.8, 38.8, and 44.9%, respectively. Additionally, three regions, SSR71, SSR6, and SDR10 were observed to be of lower importance in the country-of-origin model (SSR71: 13.2%, SSR6: 13.8%, and SDR10: 10.0%) but jumped in importance within the host-species models (SSR71: 25.3%, SSR6: 31.4%, and SDR10: 24.9%). SSR28 and SSR85 were the sole predictors to decrease in importance as the scale narrowed between the models, but this decrease was modest, especially for SSR28 (29.3 to 27.2%; Figure 6).

#### Gene functions across the geographic, sub-population, and host-species models

We observed that the genes within genomic regions that increased (SSR53, SSR42, SSR6, SSR71, SDR10) or decreased in importance (SSR28, SSR85) across the different scales contained general functions that impact virulence and immunogenicity (*phoP* and *phoR*), anaerobic survival through nitrate reduction (*narG*, *narH*, *narJ*, and *narI*), membrane structure (*pks5*, *papA4*, and *fadD25*), and ESX-3 secretion (*ecca3*, *eccb3*, *eccc3*, *esxG*, *esxH*, *espg3*, and *eccd3*). Of particular interest, the ESX-1 Type VII secretion system was identified as a unique selective sweep target in the sub-population model (*esxB*, *esxA*, and *espI*; Supplementary Table 6).

#### Regions impacted by geographic stratification of species

**Figure 7:**
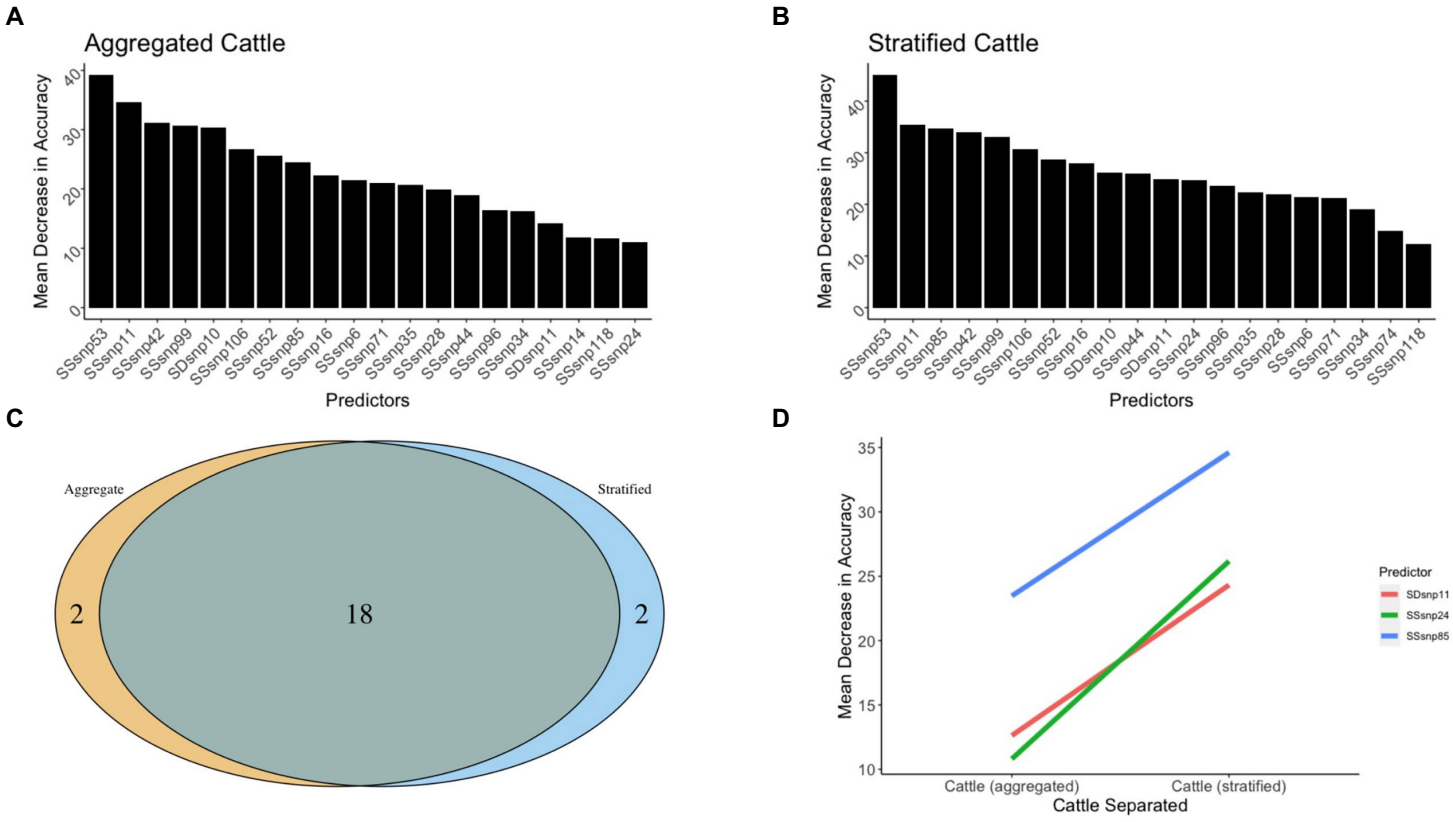
The 20 top predictors for cattle related random forest models. The 20 top predictors were shown for the **(A)** aggregated cattle and **(B)** stratified cattle random forest models. **(C)** A Venn diagram to identify regions that were shared and unique across models. **(D)** Three genomic regions between the cattle vs. wildlife models that increase or decrease model accuracy as the scales move from aggregated to stratified.

The models for determining the segregation between worldwide cattle and wildlife both possessed adequate accuracy, with the aggregated cattle model being 88% accurate in differentiating cattle from wildlife, while the stratified cattle model was 70, 83, and 94% accurate when classifying cattle from the USA, NZ, and UK, respectively, (Figures 7A,B). Between the two models, a total of 18 regions were shared as being the top 20 predictors of isolates originating in cattle versus wildlife (Figure 7C). Only two regions were found to be unique for each model. We recorded the top predictors with an absolute MDA change between the two models of at least 10 and found that all three predictors increased in importance from the aggregated cattle model compared to the stratified cattle model (Figure 7D). Of the predictors, the majority (2 out of 3) were SSRs. The only SDR noted with an absolute MDA change was SDR11. SSR24 was recorded as having the highest difference between the models with a 13.6% increase in importance (11% in the aggregated cattle model versus 24.7% in the stratified cattle model; Supplementary Table 6).

#### Gene functions that define core cattle classification

Our results of the differences in genomic region importance for the aggregated cattle vs. stratified cattle models highlighted that for global identification of cattle vs. wildlife, evolution in genes impacting the mce4 operon, which influences cholesterol utilization and intracellular survival were unique to the aggregated cattle model (*mce4D*, *mce4C*, *mce4B*, *mce4A*, *yrbE4B*, and *yrbE4A*). When isolates were classified to identify the geographically separated cattle vs. wildlife, toxin-antitoxin system functions appeared to be uniquely important to make the distinction (*vapc12* and *vapb12*). Additionally, it was observed that mycobactin biosynthesis function (*mbtH*, *mbtG*, *mbtF*, *mbtE*, and *mbtD*), as well as toxin-antitoxin function (*vapc7*, *vapb7*, *vapb8*, and *vapc8*) increased in importance when differentiating global cattle vs. geographically stratified cattle.

#### Regions that differentiate a wildlife badger from a cattle host

**Figure 8:**
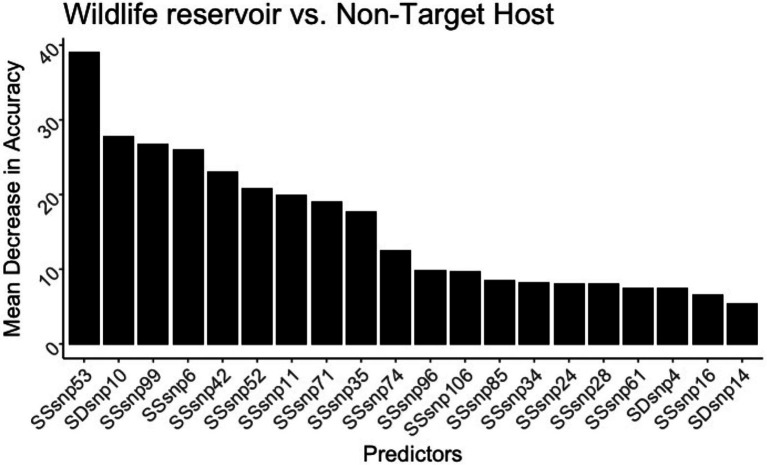
The 20 top predictors for the United Kingdom badger versus cattle random forest models. SNP evolution occurring in SSR53 is the top predictor in differentiating the species in the United Kingdom. SDR10 increased substantially in model importance, indicating its ability to differentiate badger from cattle.

In the random forest model differentiating *M. bovis* isolated from UK Badger vs. UK Cattle, the model was accurate in classifying isolates extracted from badgers at 94.6% and isolates extracted from cattle at 94.5% (Table 1). The top predictor of host in the wildlife badger and cattle random forest model was SSR53, and the other predictors were dominated by SSRs with very few SDRs (Figure 8). However, SDR10 was found to be the second highest important predictor in this model, and compared to every other computed random forest model, was the highest SDR model importance seen yet (Supplementary Table 6).

**Table 1:** Summary of the random forest classification accuracy for each developed model.

Model	Grouping	Accuracy
Country of origin	United States of America	1
	United Kingdom	0.996
	New Zealand	0.996
Population cluster	Cluster 1	1
	Cluster 2	1
	Cluster 3	0.972
	Cluster 4	1
	Cluster 5	1
	Cluster 6	1
	Cluster 7	1
	Cluster 8	0.989
Host species	UK Badger	0.946
	UK Cattle	0.945
	USA Cattle	0.8
	USA Elk	0
	USA White tailed Deer	1
	New Zealand Ferret	0.05
	New Zealand Porcine	0
	New Zealand Possum	0.11
	New Zealand Cattle	0.894
	New Zealand Cervine	0
Aggregated cattle	Bovine	0.868
	Wildlife	0.739
Stratified cattle	UK Bovine	0.937
	USA Bovine	0.7
	New Zealand Bovine	0.830
	Wildlife	0.772
UK Badger and Cattle	Badger	0.946
	Cattle	0.945

Accuracy is defined as the proportion of correct classifications of a grouping over the total number of attempts. Values can range from 0 to 1.

## Discussion

This analysis presents one of the first studies in *Mycobacterium bovis* research that uses comparative genomics and machine learning approaches to identify putative genomic factors that contribute to across-scale evolution of *M. bovis* isolates. Using publicly available *M. bovis* sequences from well characterized molecular epidemiological studies, we investigated specific genomic signatures across ecological scales such as geographical locations, host-species, and pathogen population structure. To best identify these genomic signatures, we used a probabilistic framework to identify regions possessing high SNP counts and/or regions with high evidence for selective sweeps. After, we used random forest models to assess the importance of these genomic regions and other genomic variables such as GC content and the number of INDELS in classifying the *M. bovis* genomics across the three different scales. The combined application of machine learning and comparative genomics of pathogenic organisms to better understand connections between evolution and molecular epidemiology is still in its early stages. For *M. bovis* research, machine learning techniques were first explored in the work by Crispell et al. (2019), where epidemiological metrics were used to investigate their contribution to the genomic similarity of isolates during an *M. bovis* outbreak in cattle and badger populations. Likewise, there have been numerous other studies that determined specific *M. bovis* evolutionary targets that coincide with various lineages (Patané et al., 2017; Zimpel et al., 2020).

The random forest models we developed were helpful in investigating which genomic regions became more important as ecological scales changed. In both the Across-Scales model comparison and the Aggregated *vs*. Stratified cattle model, pathogenic genes were harbored within the genomic regions that had sharp increases or decreases when the predictor importance was compared between models. While the genomic regions importance is not an assertion of being directly related to host adaptation, it provides an early *in-silico* examination of genes that possibly could play a role in adaptation to new environments. We hope that by identifying and reporting these genes, researchers that study *M. bovis* pathogenesis can further elucidate how the functions we highlighted impact *M. bovis* transmission in the very common multi-host environments.

Across every single model, SSRs were the most consistently important predictor in differentiating isolates from different geographic, sub-population, or host species groupings. Amongst the top 20 predictors in every individual model, SSRs had very high MDAs and achieved high levels of importance, despite the presence of SDRs in the full data. This indicated that evolution in selective sweep regions play a major role in differentiating isolates across the different scales. This would match what is observed in other pathogenic bacteria that maintain multiple host-associated clonal lineages such as *Campylobacter jejuni*, *Staphylococcus aureus* and *Salmonella enterica* (Sheppard et al., 2018). Since *M. bovis* evolution is also described as evolving clonally, the main avenue of adapting to new host populations would likely be achieved predominantly through mutations that confer high selective advantage within a particular niche. Monitoring the genomic regions with SNPs in high linkage disequilibrium could be leveraged to identify genes that are essential for *M. bovis* transmission between different ecological scales and perhaps provide a signal that *M. bovis* is being maintained within a new population.

While SSRs were predominant amongst the models, a few SDRs did rank as the top 20 most important predictors (SDR3, SDR9, SDR10, SDR11, and SDR14). In the UK badger and cattle model, SDR10 was the second highest predictor of wildlife badger status and additionally had the highest importance rank amongst all the SDRs. SDR10 contains genes that are implicated in mycobacterial adaptation (*vapb18* and *vapc18*) as well as lipoproteins that affect lipid modifications (*lppA*, *lppB*, and *lprR*). Elevated mutation rates in these genes could have a direct impact on adaptation to the immunological pressures present within the UK wildlife reservoir through changes in membrane structure. Additionally, since genes within the SDR10 region gained high importance in the badger and cattle model, genomic regions of high SNP density might play a larger role in classifying local isolates than global ones. Altogether, this provides some indication that while selective sweep sites are important genomic signatures to investigate *M. bovis* evolution, the contribution of genomic regions that undergo excessive mutation should be further investigated. The accuracy of the random forest models to classify country-of-origin and sub-population groups was consistently high, but in the case of the host-species model, the model failed in classifying correctly all the host-species, having just a few adequately classified. The reasons for the poor performance of the host-species model in certain host-species groups could be due to lack of host species structure along the phylogeny. When compared to the number of SNPs within key SSRs, the SNP count metric correlated poorly with host species (Supplementary Figures 4–6). We analyzed the top three SSRs (SSR53, SSR85, and SSR11) that were used to classify host species and Supplementary Figures 4–6 show that in clades with low host-population structure, the number of SNPs in these regions did not have enough resolution to distinguish host-species within clades. Despite this poor accuracy, the utilization of sub-populations, first described by Rodrigues et al. (2021) to define *M. bovis* in Brazil, sub-divided into eight distinct population clusters, showed remarkable accuracy for correctly identifying the proper sub-population. The inferred population clusters encompassed *M. bovis* genomic similarity isolated from multiple host species, so this method is most likely overcoming the accuracy problem found in the host species model by defining communities of hosts that share a similar evolutionary history. Since our results show high accuracy in differentiating the samples based on their fastBAPS derived clusters rather than host species, future studies should further explore the relationship between population clusters, transmission, and selective sweeps in order to dissect *M. bovis* adaptation. Furthermore, these results suggest that when researchers investigate the genomic factors that contribute to *M. bovis* adaptation, it is more suitable to conduct these comparative analyses at local scales rather than global scales (where evolution occurred separately for a prolonged time between distinct regions). Otherwise, comparisons made between isolates based solely on geographic distance has the potential to mask biologically relevant results that are contributing to putative adaptation.

One limitation of this study was the amount of data available for each host-species in the different geographical locations. The data were sparse regarding certain host-species populations, making it difficult to draw conclusions about the model’s ability to differentiate host species populations. In terms of accuracy, we noticed that low accuracy classes typically were not heavily sampled, but exceptions also existed. For example, 5 USA elk were never predicted accurately in our species model, while 13 USA cattle were correctly classified 80%. It appears that sub-population inference might be the best approach to mitigate the classification issue and identify variation occurring amongst *M. bovis* isolates, but without proper sampling of particular host-species it will be difficult to conclude if the low accuracy in classifying species is due to factors other than sample size. Additionally, we focused on selective sweep sites and SNP dense regions as the main genomic signatures of interest, and while our analyses produced insights regarding the relationship between *M. bovis* genomic evolution and ecological niche membership, there are other genomic signatures that could be investigated further. The data for this analysis was based on the number of SNPs within a SDR or SSR, but information about the exact SNPs that provided statistical support to differentiate *M. bovis* amongst various niches was not investigated. In future work, specialized bacterial genome-wide association studies (bacGWAS) would be useful to find influential SNPs, and furthermore answer the question of how these mutations impact the structure of particular genes (Lees et al., 2018).

## Data availability statement

The WGS datasets and corresponding metadata analysed during the current study were downloaded from the NCBI Sequence Read Archive under the Bioproject Accession numbers: PRJNA363037 (NZ), PRJNA523164 (UK), and PRJNA251692 (USA). The scripts used for this publication are freely available on the following link: https://github.com/salvadorlab/LegallSalvador2022_MbovisAcrossScales.

## Author contributions

NL designed the study, analyzed data, and wrote first draft of manuscript. LCMS designed the study, supervised data analysis, contributed to study interpretation, and revised the manuscript. All authors contributed to the article and approved the submitted version.

## Funding

This work was supported by the National Science Foundation under grant no. DGE-1545433 501 to NL and startup funds to LS from the University of Georgia Office of Research.

## Conflict of interest

The authors declare that the research was conducted in the absence of any commercial or financial relationships that could be construed as a potential conflict of interest.

## Publisher’s note

All claims expressed in this article are solely those of the authors and do not necessarily represent those of their affiliated organizations, or those of the publisher, the editors and the reviewers. Any product that may be evaluated in this article, or claim that may be made by its manufacturer, is not guaranteed or endorsed by the publisher.
