# Supplementary material for: Selective sweep sites and SNP dense regions differentiate Mycobacterium bovis isolates across scales
Source: Front Microbiol. 2022 Sep 7;13:787856. doi: 10.3389/fmicb.2022.787856 (PMC9489834; doi:10.3389/fmicb.2022.787856)
Supplement: Supplementary file 1 [file Data_Sheet_1.ZIP › SupplementaryMaterials_Revised/SupplementaryMaterials_Revised.docx]

Supplementary Material

# Supplementary Data

**Supplementary File 1.** *Mycobacterium bovis* SNP alignment in FASTA format. This FASTA alignment was used to generate the phylogenetic results and determine the sub-populations present in the dataset.

**Supplementary File 2.** A summary file of the IQ-TREE inference process for the original 700 *M. bovis* alignment. In this file we can find information related to Model Testing, Model parameter estimation, phylogenetic inference, and bootstrap support calculation.

**Supplementary File 3.** A list of the genomic regions and general genomic metrics that were removed from the analyses (before the random forest modeling) due to high correlation with the other predictors.

# Supplementary Figures


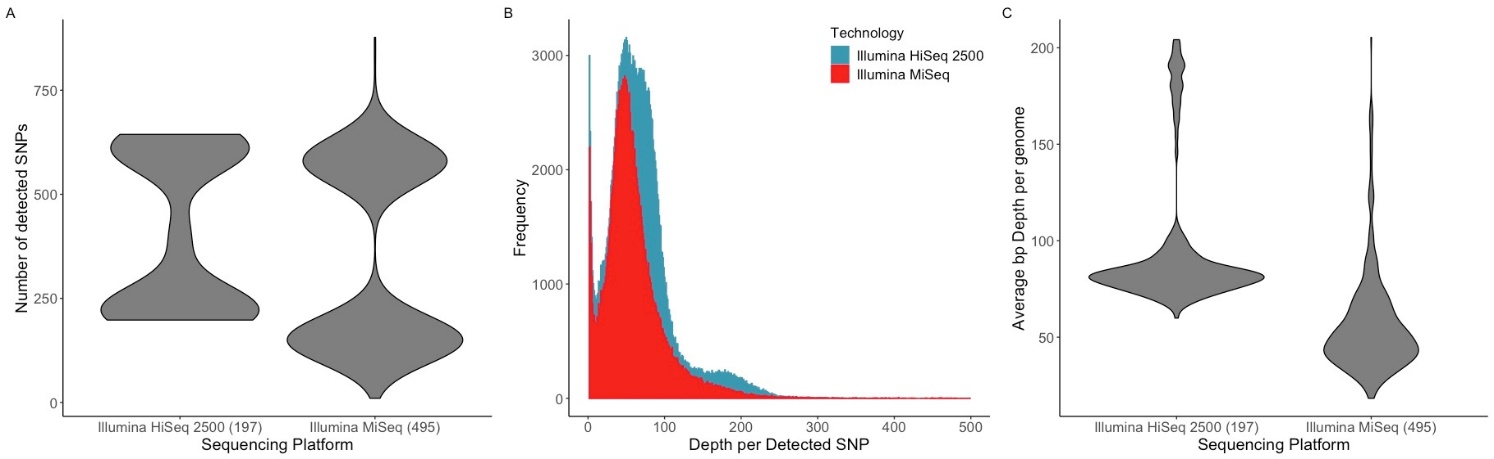
 **Supplementary Figure 1. Bioinformatic analysis results by sequencing platform.** (A) The total number of SNPs detected in 692 *Mycobacterium bovis* isolates sequenced by Hiseq and MiSeq platforms. (B) Distribution of read depth at a detected SNP position by sequencing platform. The red and blue distributions represent SNPs identified on isolates sequenced with the MiSeq and HiSeq platforms, respectively. (C) The average depth at each base pair of isolates sequenced by HiSeq and MiSeq sequencing platforms. A total of 8 isolates sequenced by NextSeq were not included in the figure.


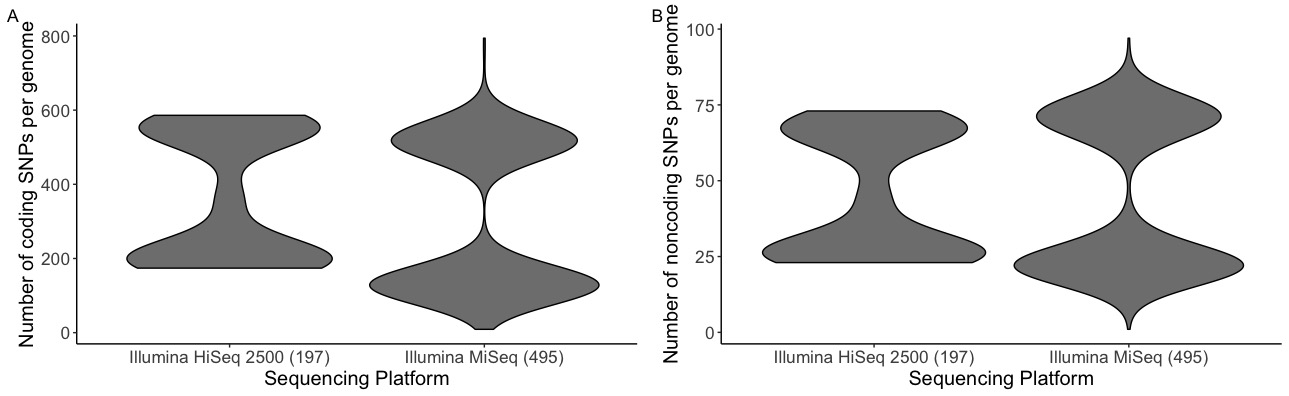
**Supplementary Figure 2.** **The number of SNPs per genome by sequencing platform.** The number of SNPs found in (A) coding and (B) non-coding regions of the *Mycobacterium bovis* genome, calculated based on the sequencing platform that was used. Isolates sequenced on a NextSeq platform (8) were not included in the violin plots due to insufficient number of isolates.


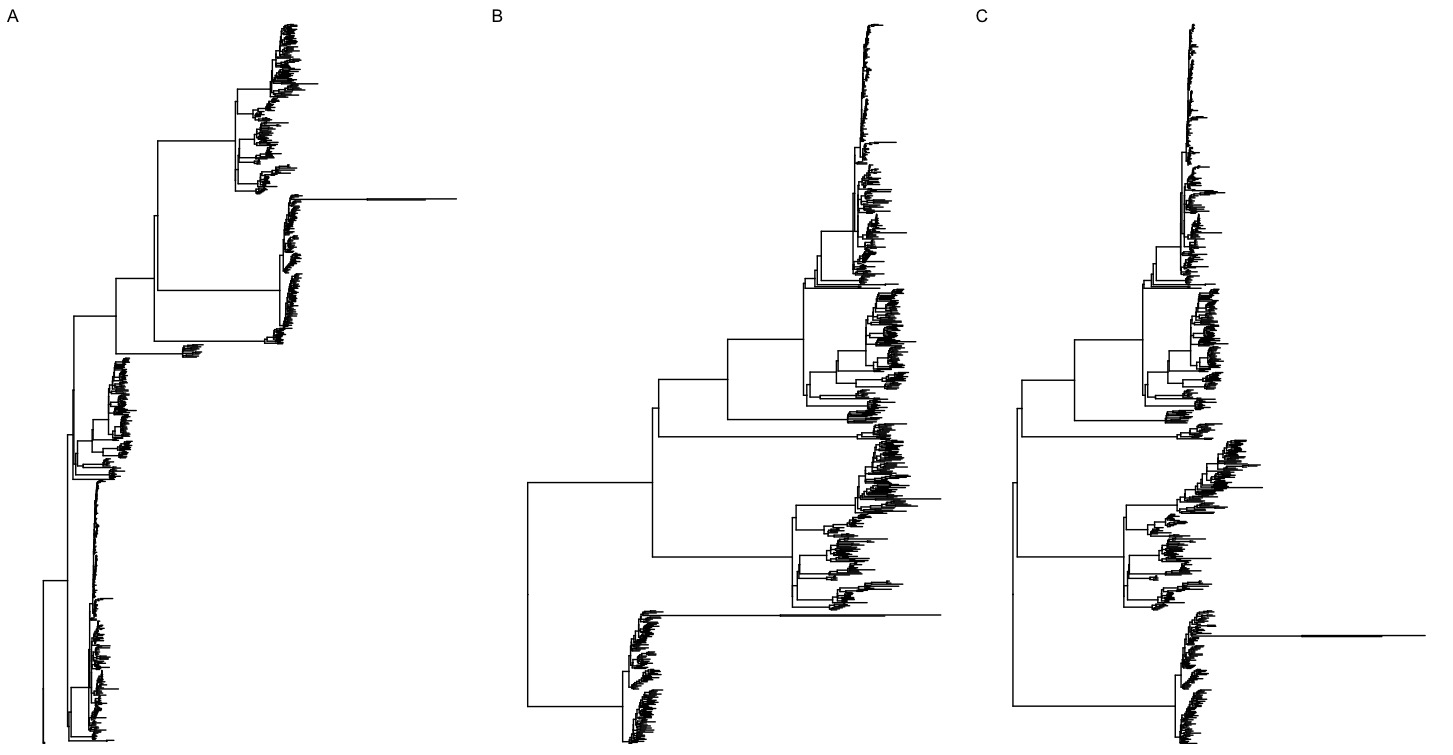


**Supplementary Figure 3. The phylogeny of *Mycobacterium bovis* isolates with different rooting strategies.** Each phylogenetic tree represents all of the 700 *M. bovis* isolates, but was rooted by a different mechanism: (A) *M. bovis* reference outgroup AF2122/97, (B) Midpoint rooting, and (C) Minimal Ancestor Deviation rooting.

A B


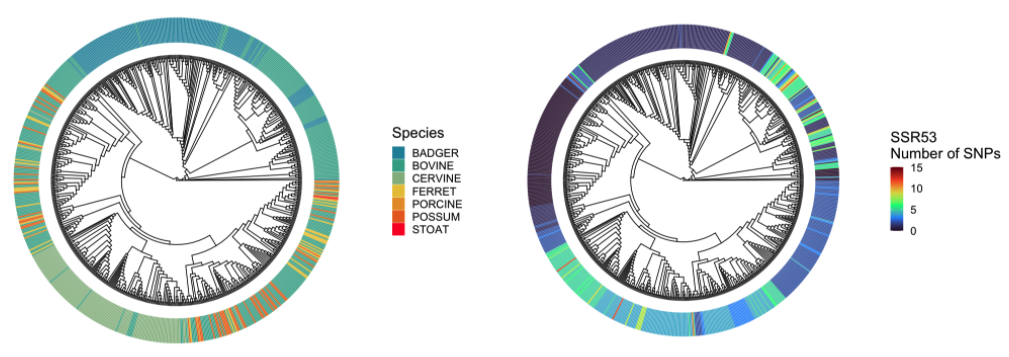


**Supplementary Figure 4.** **Distribution of host-species and number of SNPs within the *Mycobacterium bovis* SSR53 region.** *M. bovis* phylogeny of 700 isolates colored by (A) host-species and by the (B) number of SNPs within the SSR53 region.

A B


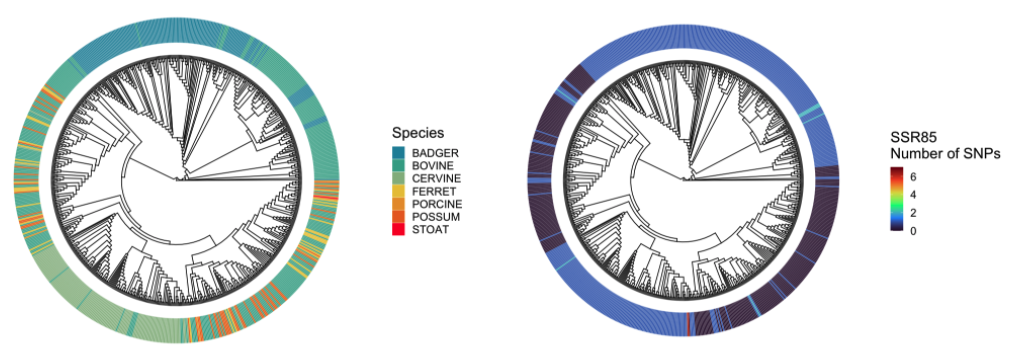


**Supplementary Figure 5.** **Distribution of host-species and number of SNPs within the *Mycobacterium bovis* SSR85 region.** *M. bovis* phylogeny of 700 isolates colored by (A) host-species and by the (B) number of SNPs within the SSR85 region.


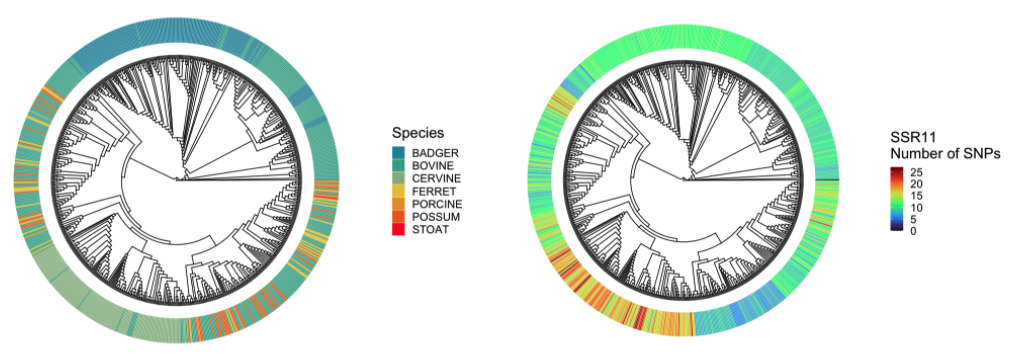


**Supplementary Figure 6.** **Distribution of host-species and number of SNPs within the *Mycobacterium bovis* SSR11 region.** *M. bovis* phylogeny of 700 isolates colored by (A) host-species and by the (B) number of SNPs within the SSR11 region.

## Supplementary Tables

**Supplementary Table 1. Description of the data used in this study**. Description of metadata, bioinformatic analysis sequencing statistics, project and sample information of all FASTQ files downloaded from the NCBI Sequence Read Archives. For each genome, we recorded the following information: Sample SRA Accession ID, Country, Species, Bioproject, Biosample, R1, R2, Average Coverage, Genome Coverage, Average Read Length, Number of detected SNPs, Number of coding SNPs, Number of non-coding SNPs, Average base quality, Average bp depth per genome, Average mapping quality, and sequencing technology.

**Supplementary Table 2.** **Phylogenetic tree comparison.** A comparison of the phylogeny created from the original data was performed to the one produced by Gubbins. Both trees were inferred using IQ-TREE and inferred with the same TIM2+F+ASC+R4 model. The trees were compared unrooted using the Robinson Foulds distance (the number of topological changes necessary to change one tree to another), and the percentages of nodes with over 50% and 75% support are shown below.

|  | Original Tree | Gubbins Tree |
| --- | --- | --- |
| Robinson Foulds Distance | 592 | |
| % nodes bootstrap above 50 | 81.7 | 88.7 |
| % nodes bootstrap above 75 | 70.1 | 75.7 |

**Supplementary Table 3.** **Phylogenetic topologies comparison when differing rooting strategies**. A Robinson Foulds Distance was computed between each pair of phylogenetic trees rooted with three different methods: using the *Mycobacterim bovis* reference genome as an Outgroup, Midpoint rooting, and Minimal Ancestor Deviation (MAD).

|  | Outgroup | Midpoint | MAD |
| --- | --- | --- | --- |
| Outgroup | 0 | 14 | 12 |
| Midpoint | 14 | 0 | 0 |
| MAD | 12 | 0 | 0 |

**Supplementary Table 4. Genomic coordinates of SNP Dense Regions (SDRs).** The regions found in the *Mycobacterium bovis* genome that contained a highly significant number of SNPs within a 1,000 bp window.

| Region ID | Start | End | Fragment Length | Number of Coding Sequences |
| --- | --- | --- | --- | --- |
| SDR1 | 416350 | 418600 | 2250 | 3 |
| SDR2 | 964500 | 966350 | 1850 | 3 |
| SDR3 | 1276850 | 1278750 | 1900 | 2 |
| SDR4 | 1707150 | 1708200 | 1050 | 1 |
| SDR5 | 1985800 | 1987450 | 1650 | 1 |
| SDR6 | 2192350 | 2194000 | 1650 | 2 |
| SDR7 | 2282400 | 2285250 | 2850 | 2 |
| SDR8 | 2287500 | 2289400 | 1900 | 1 |
| SDR9 | 2293550 | 2295500 | 1950 | 1 |
| SDR10 | 2837450 | 2840100 | 2650 | 6 |
| SDR11 | 3079950 | 3081350 | 1400 | 1 |
| SDR12 | 3207700 | 3209500 | 1800 | 1 |
| SDR13 | 3776650 | 3777750 | 1100 | 1 |
| SDR14 | 4293250 | 4295000 | 1750 | 3 |

**Supplementary Table 5. Genomic coordinates of Selective Sweep Regions (SSRs).** The regions found in the *Mycobacterium bovis* genome that contained significant evidence for the presence of a selective sweep site.

| Region ID | Start | End | Fragment Length | Number of Coding Sequences |
| --- | --- | --- | --- | --- |
| SSR1 | 7705 | 37715 | 30010 | 33 |
| SSR2 | 39025 | 62944 | 23919 | 23 |
| SSR3 | 66864 | 79474 | 12610 | 9 |
| SSR4 | 95573 | 108183 | 12610 | 12 |
| SSR5 | 116453 | 129933 | 13480 | 7 |
| SSR6 | 153862 | 165602 | 11740 | 12 |
| SSR7 | 190402 | 205622 | 15220 | 15 |
| SSR8 | 213021 | 224761 | 11740 | 13 |
| SSR9 | 229551 | 244771 | 15220 | 13 |
| SSR10 | 264350 | 288270 | 23920 | 21 |
| SSR11 | 309589 | 362217 | 52628 | 39 |
| SSR12 | 391366 | 426595 | 35229 | 32 |
| SSR13 | 447914 | 484013 | 36099 | 35 |
| SSR14 | 495762 | 505762 | 10000 | 10 |
| SSR15 | 512291 | 533601 | 21310 | 19 |
| SSR16 | 542741 | 573621 | 30880 | 36 |
| SSR17 | 578411 | 596241 | 17830 | 20 |
| SSR18 | 628001 | 638001 | 10000 | 11 |
| SSR19 | 639311 | 654531 | 15220 | 17 |
| SSR20 | 654971 | 674541 | 19570 | 19 |
| SSR21 | 687161 | 704121 | 16960 | 22 |
| SSR22 | 708911 | 723261 | 14350 | 18 |
| SSR23 | 728051 | 753711 | 25660 | 30 |
| SSR24 | 756761 | 773721 | 16960 | 14 |
| SSR25 | 785471 | 796341 | 10870 | 12 |
| SSR26 | 805481 | 818091 | 12610 | 21 |
| SSR27 | 819401 | 836361 | 16960 | 21 |
| SSR28 | 850721 | 863331 | 12610 | 16 |
| SSR29 | 872471 | 886821 | 14350 | 15 |
| SSR30 | 905531 | 926841 | 21310 | 30 |
| SSR31 | 954251 | 981651 | 27400 | 27 |
| SSR32 | 989051 | 1011231 | 22180 | 20 |
| SSR33 | 1026461 | 1048641 | 22180 | 22 |
| SSR34 | 1054301 | 1075611 | 21310 | 20 |
| SSR35 | 1080401 | 1091271 | 10870 | 9 |
| SSR36 | 1094321 | 1135641 | 41320 | 43 |
| SSR37 | 1137821 | 1154781 | 16960 | 15 |
| SSR38 | 1163921 | 1191321 | 27400 | 31 |
| SSR39 | 1197851 | 1207851 | 10000 | 10 |
| SSR40 | 1221341 | 1231341 | 10000 | 10 |
| SSR41 | 1257881 | 1287021 | 29140 | 31 |
| SSR42 | 1289201 | 1300071 | 10870 | 7 |
| SSR43 | 1309211 | 1320081 | 10870 | 6 |
| SSR44 | 1346621 | 1357491 | 10870 | 12 |
| SSR45 | 1375331 | 1385331 | 10000 | 11 |
| SSR46 | 1394471 | 1423611 | 29140 | 25 |
| SSR47 | 1439711 | 1461021 | 21310 | 21 |
| SSR48 | 1465811 | 1494951 | 29140 | 26 |
| SSR49 | 1540631 | 1556721 | 16090 | 17 |
| SSR50 | 1605881 | 1615881 | 10000 | 9 |
| SSR51 | 1618931 | 1628931 | 10000 | 9 |
| SSR52 | 1665911 | 1682871 | 16960 | 18 |
| SSR53 | 1697231 | 1724631 | 27400 | 19 |
| SSR54 | 1725941 | 1739421 | 13480 | 12 |
| SSR55 | 1762481 | 1779441 | 16960 | 24 |
| SSR56 | 1779881 | 1795101 | 15220 | 17 |
| SSR57 | 1800761 | 1812501 | 11740 | 11 |
| SSR58 | 1819901 | 1830771 | 10870 | 8 |
| SSR59 | 1837301 | 1852521 | 15220 | 13 |
| SSR60 | 1862531 | 1878621 | 16090 | 6 |
| SSR61 | 1916471 | 1941261 | 24790 | 30 |
| SSR62 | 1949531 | 1968231 | 18700 | 19 |
| SSR63 | 1981721 | 1991721 | 10000 | 7 |
| SSR64 | 1999991 | 2009991 | 10000 | 11 |
| SSR65 | 2020871 | 2031741 | 10870 | 12 |
| SSR66 | 2055671 | 2067411 | 11740 | 10 |
| SSR67 | 2085251 | 2101341 | 16090 | 19 |
| SSR68 | 2101781 | 2112651 | 10870 | 10 |
| SSR69 | 2133971 | 2145711 | 11740 | 16 |
| SSR70 | 2182691 | 2199651 | 16960 | 25 |
| SSR71 | 2205311 | 2237061 | 31750 | 31 |
| SSR72 | 2242721 | 2274471 | 31750 | 33 |
| SSR73 | 2275781 | 2315361 | 39580 | 30 |
| SSR74 | 2316671 | 2329281 | 12610 | 17 |
| SSR75 | 2335811 | 2351031 | 15220 | 12 |
| SSR76 | 2367131 | 2382351 | 15220 | 16 |
| SSR77 | 2383661 | 2427591 | 43930 | 44 |
| SSR78 | 2428901 | 2439771 | 10870 | 11 |
| SSR79 | 2446301 | 2471091 | 24790 | 23 |
| SSR80 | 2483711 | 2494581 | 10870 | 18 |
| SSR81 | 2503721 | 2518071 | 14350 | 16 |
| SSR82 | 2558531 | 2570271 | 11740 | 17 |
| SSR83 | 2577671 | 2587671 | 10000 | 11 |
| SSR84 | 2592461 | 2605941 | 13480 | 12 |
| SSR85 | 2622041 | 2640741 | 18700 | 14 |
| SSR86 | 2645531 | 2656401 | 10870 | 9 |
| SSR87 | 2659451 | 2697291 | 37840 | 42 |
| SSR88 | 2698601 | 2746881 | 48280 | 49 |
| SSR89 | 2784731 | 2798211 | 13480 | 12 |
| SSR90 | 2801261 | 2819961 | 18700 | 13 |
| SSR91 | 2829971 | 2846061 | 16090 | 24 |
| SSR92 | 2896091 | 2909571 | 13480 | 19 |
| SSR93 | 2916971 | 2926971 | 10000 | 13 |
| SSR94 | 2985701 | 2995701 | 10000 | 11 |
| SSR95 | 3004841 | 3014841 | 10000 | 11 |
| SSR96 | 3015281 | 3027021 | 11740 | 13 |
| SSR97 | 3030071 | 3045291 | 15220 | 22 |
| SSR98 | 3051821 | 3067911 | 16090 | 17 |
| SSR99 | 3072701 | 3090531 | 17830 | 27 |
| SSR100 | 3092711 | 3128811 | 36100 | 36 |
| SSR101 | 3139691 | 3194931 | 55240 | 55 |
| SSR102 | 3195371 | 3223641 | 28270 | 14 |
| SSR103 | 3257141 | 3290631 | 33490 | 29 |
| SSR104 | 3298031 | 3308901 | 10870 | 10 |
| SSR105 | 3345881 | 3357621 | 11740 | 13 |
| SSR106 | 3367631 | 3391551 | 23920 | 22 |
| SSR107 | 3391991 | 3416781 | 24790 | 25 |
| SSR108 | 3434621 | 3444621 | 10000 | 14 |
| SSR109 | 3526841 | 3536841 | 10000 | 8 |
| SSR110 | 3538151 | 3568161 | 30010 | 36 |
| SSR111 | 3587741 | 3599481 | 11740 | 13 |
| SSR112 | 3654731 | 3669951 | 15220 | 21 |
| SSR113 | 3725201 | 3759561 | 34360 | 37 |
| SSR114 | 3777401 | 3790881 | 13480 | 14 |
| SSR115 | 3796541 | 3806541 | 10000 | 13 |
| SSR116 | 3806981 | 3818721 | 11740 | 12 |
| SSR117 | 3822641 | 3836991 | 14350 | 18 |
| SSR118 | 3862661 | 3873531 | 10870 | 11 |
| SSR119 | 3904421 | 3917901 | 13480 | 13 |
| SSR120 | 3936611 | 3960531 | 23920 | 24 |
| SSR121 | 3966191 | 3976191 | 10000 | 11 |
| SSR122 | 4004471 | 4028391 | 23920 | 28 |
| SSR123 | 4029701 | 4044921 | 15220 | 17 |
| SSR124 | 4060151 | 4075371 | 15220 | 17 |
| SSR125 | 4075811 | 4085811 | 10000 | 12 |
| SSR126 | 4106261 | 4131921 | 25660 | 21 |
| SSR127 | 4137581 | 4176291 | 38710 | 50 |
| SSR128 | 4185431 | 4195431 | 10000 | 9 |
| SSR129 | 4222841 | 4234581 | 11740 | 9 |
| SSR130 | 4248941 | 4259811 | 10870 | 13 |
| SSR131 | 4280261 | 4311141 | 30880 | 25 |
| SSR132 | 4334201 | 4348551 | 14350 | 14 |

**Supplementary Table 6. Genes residing in important genomic regions by model used in the Random Forest Analysis.** For each model, we recorded which genes were nested within uniquely identified genomic regions that were only found to be a top twenty predictor within their own model, and interesting genomic regions whose importance in the model increased/decreased when compared across models (i.e. Across Scales and Aggregated vs. Stratified).

| Model | Unique/Interesting Regions | Genes |
| --- | --- | --- |
| Country | SSR118 | mce4F |
|  |  | lprN |
|  |  | mce4D |
|  |  | mce4C |
|  |  | mce4B |
|  |  | mce4A |
|  |  | yrbE4B |
|  |  | yrbE4A |
|  |  | fdxD |
|  |  | fadE26 |
|  | SSR17 | gpm1 |
|  |  | senX3 |
|  |  | regX3 |
|  |  | ppx1 |
|  |  | proC |
|  |  | galE2 |
|  |  | cmaA2 |
| sSpecies | SSR35 | ctpV |
|  |  | echA7 |
|  |  | fadE12 |
|  |  | accA2 |
|  |  | accD2 |
|  |  | fadE13 |
|  |  | PE_PGRS16 |
|  | SDR10 | lppA |
|  |  | lprR |
|  |  | lppB |
|  |  | vapb18 |
|  |  | vapc18 |
| Population Cluster | SDR14 | esxB |
|  |  | esxA |
|  |  | espi |
| Across Scales | SSR28 | PPE12 |
|  |  | phoP |
|  |  | phoR |
|  |  | adhB |
|  |  | cyp51 |
|  |  | cyp123 |
|  |  | aldA |
|  | SSR42 | narG |
|  |  | narH |
|  |  | narJ |
|  |  | narI |
|  |  | typA |
|  |  | lpqW |
|  | SSR71 | mpt64 |
|  |  | nrdF1 |
|  |  | vapc36 |
|  |  | vapB36 |
|  |  | PE_PGRS35 |
|  |  | cfp21 |
|  |  | erm(37) |
|  |  | mazf6 |
|  |  | mazE6 |
|  |  | ctpG |
|  |  | cmtr |
|  |  | ctpF |
|  |  | fabG3 |
|  |  | otsB1 |
|  | SSR53 | fadD25 |
|  |  | mmpL12 |
|  |  | wbbL2 |
|  |  | pks5 |
|  |  | papA4 |
|  |  | fadD24 |
|  |  | adh |
|  | SSR6 | treS |
|  |  | mak |
|  |  | fbpC |
|  |  | htdz |
|  |  | fadE1 |
|  |  | fgd2 |
|  |  | cyp138 |
|  |  | msrA |
|  | SSR11 | narK3 |
|  |  | aac |
|  |  | oplA |
|  |  | narU |
|  |  | fadD2 |
|  |  | fadE6 |
|  |  | vapc25 |
|  |  | PE_PGRS3a |
|  |  | PE_PGRS3 |
|  |  | PE_PGRS4 |
|  |  | PPE3 |
|  |  | ecca3 |
|  |  | eccb3 |
|  |  | eccc3 |
|  |  | PE5 |
|  |  | PPE4 |
|  |  | esxG |
|  |  | esxH |
|  |  | espg3 |
|  |  | eccd3 |
|  |  | mycp3 |
|  |  | ecce3 |
|  |  | tam |
|  |  | stf0 |
|  | SDR10 | lppA |
|  |  | lprR |
|  |  | lppB |
|  |  | vapb18 |
|  |  | vapc18 |
| Aggregated | SSR14 | ackA |
|  |  | pknG |
|  |  | glnH |
|  |  | mutT3 |
|  |  | thiE |
|  |  | thiO |
|  |  | thiS |
|  |  | thiG |
|  |  | lpqL |
|  | SSR118 | mce4F |
|  |  | lprN |
|  |  | mce4D |
|  |  | mce4C |
|  |  | mce4B |
|  |  | mce4A |
|  |  | yrbE4B |
|  |  | yrbE4A |
|  |  | fdxD |
|  |  | fadE26 |
| Stratified | SSR61 | cycA |
|  |  | PPE22 |
|  |  | PPE23 |
|  |  | scpa |
|  |  | scpb |
|  |  | cmk |
|  |  | engA |
|  |  | fadB3a |
|  |  | fadB3b |
|  |  | vapc12 |
|  |  | vapb12 |
|  | SSR74 | cobM |
|  |  | cobLb |
|  |  | cobLa |
|  |  | lppJ |
| Aggregated vs. Stratified | SSR85 | phoH1 |
|  |  | PE_PGRS40 |
|  |  | rsmE |
|  |  | dnaJ2 |
|  |  | hrcA |
|  |  | cfp2 |
|  |  | mbtH |
|  |  | mbtG |
|  |  | mbtF |
|  |  | mbtE |
|  |  | mbtD |
|  | SSR24 | mazf2 |
|  |  | maze2 |
|  |  | vapc7 |
|  |  | vapb7 |
|  |  | atsD |
|  |  | vapb8 |
|  |  | vapc8 |
|  |  | rpoB |
|  |  | rpoC |
|  |  | end |
|  |  | lpqP |
|  |  | fadE8 |
| Reservoir | SDR10 | lppA |
|  |  | lprR |
|  |  | lppB |
|  |  | vapb18 |
|  |  | vapc18 |
